# Down-regulation of MKP-1 in hippocampus protects against stress-induced depression-like behaviors and neuroinflammation

**DOI:** 10.1038/s41398-024-02846-7

**Published:** 2024-03-01

**Authors:** Mengjun Geng, Qiujing Shao, Jiacheng Fu, Jingyang Gu, Laipeng Feng, Liqin Zhao, Cong Liu, Junlin Mu, Xiaoli Zhang, Mingjun Zhao, Xinsheng Guo, Cai Song, Yan Li, Huiying Wang, Changhong Wang

**Affiliations:** 1 The Second Affiliated Hospital of Xinxiang Medical University, Henan Mental Hospital, 453002 Xinxiang, Henan China; 2 Henan Key Laboratory of Biological Psychiatry, Xinxiang Medical University, 453002 Xinxiang, Henan China; 3 Guangdong Ocean University College of Food Science and Technoligy, Zhanjiang, China; 4 The Second Affiliated Hospital of Zhengzhou University, 450014 Zhengzhou, Henan China; 5 Henan Provincial Key Laboratory of Sleep Medicine, 453002 Xinxiang, Henan China

**Keywords:** Molecular neuroscience, Clinical genetics, Depression

## Abstract

Chronic stress is the primary environmental risk factor for major depressive disorder (MDD), and there is compelling evidence that neuroinflammation is the major pathomechanism linking chronic stress to MDD. Mitogen-activated protein kinase (MAPK) phosphatase-1 (MKP-1) is a negative regulator of MAPK signaling pathways involved in cellular stress responses, survival, and neuroinflammation. We examined the possible contributions of MKP-1 to stress-induced MDD by comparing depression-like behaviors (anhedonia, motor retardation, behavioral despair), neuroinflammatory marker expression, and MAPK signaling pathways among rats exposed to chronic unpredictable mild stress (CUMS), overexpressing MKP-1 in the hippocampus, and CUMS-exposed rats underexpressing MKP-1 in the hippocampus. Rats exposed to CUMS exhibited MKP-1 overexpression, greater numbers of activated microglia, and enhanced expressions of neuroinflammatory markers (interleukin [IL]-6, [IL]-1β, tumor necrosis factor [TNF]-ɑ, and decreased phosphorylation levels of ERK and p38 in the hippocampus as well as anhedonia in the sucrose preference test, motor retardation in the open field, and greater immobility (despair) in the forced swimming tests. These signs of neuroinflammation and depression-like behaviors and phosphorylation levels of ERK and p38 were also observed in rats overexpressing MKP-1 without CUMS exposure, while CUMS-induced neuroinflammation, microglial activation, phosphorylation levels of ERK and p38, and depression-like behaviors were significantly reversed by MKP-1 knockdown. Moreover, MKP-1 knockdown promoted the activation of the MAPK isoform ERK, implying that the antidepressant-like effects of MKP-1 knockdown may be mediated by the ERK pathway disinhibition. These findings suggested that hippocampal MKP-1 is an essential regulator of stress-induced neuroinflammation and a promising target for antidepressant development.

## Introduction

Major depressive disorder (MDD) is a common mental disorder that affects 185 million people globally [1]. The primary clinical manifestations of MDD are persistent sadness, loss of pleasure and interest (anhedonia), feelings of hopelessness and worthlessness, and in severe cases suicidal ideation, which may lead to suicide attempt [2]. Depression prevalence and suicide rates have risen further during the COVID-19 pandemic, so the elucidation of pathogenic mechanisms and treatment development is more important than ever [3, 4]. There are numerous hypotheses on the pathophysiology of MDD [5–8], but current theories have not led to broadly effective treatments for many patients.

The stress response is a necessary survival mechanism required for adaptation to environmental challenges, but persistent or repeated stress can have numerous adverse effects on physical and mental health [9]. There is compelling evidence that excessive release of inflammatory factors induced by chronic stress contributes to MDD onset and progression [10, 11]. Chronic life stress is associated with elevated levels of inflammatory markers [12] in the absence of physical injury [13]. In turn, inflammation can cause profound changes in behavior, including depressive symptoms such as sad mood, anhedonia, fatigue, psychomotor retardation, and social withdrawal [13].

Microglia are the resident macrophages of the central nervous system and critical regulators of the brain immune microenvironment [14]. Chronic psychological stress induces morphological and functional changes in microglia collectively termed activation [15]. In this activated state, microglia secrete various pro-inflammatory mediators such as interleukin[IL]-6, [IL]-1β, and tumor necrosis factor [TNF]-ɑ that promote neuroinflammation [9] and may contribute to the pathogenesis of depression [13]. This neuroimmune hypothesis of depression suggests that interventions targeting neuroinflammatory pathways may be effective antidepressant treatments.

Mitogen-activated protein kinases (MAPKs) are a group of serine/threonine kinases highly conserved in eukaryotes that function in a variety of cellular processes, including cell stress and immune responses [16]. The MAPKs can be categorized into three subfamilies, ERK, JNK, and p38, that are inactivated through dephosphorylation by specific upstream signaling factors including MAPK kinases. Conversely, a group of dual-specificity serine/threonine MAPK phosphatases (MKPs) has been identified that deactivate MAPKs. Dephosphorylation by MKPs is considered an important feedback control mechanism [17]. Among these MKPs, MKP-1 is ubiquitously expressed and regulates multiple MAPK-dependent processes including cell proliferation, differentiation, oxidative stress response, immune function, and apoptosis [18].

In recent years, it has been demonstrated that aberrant MKP-1 signaling is involved in a number of brain diseases, such as Alzheimer’s disease (AD) and Huntington’s disease (HD) [19, 20]. In addition, overactivation of MKP-1 has been implicated in inflammation-mediated neuronal dysfunction [20]. Stress results in the excessive activation of MKP-1, which in turn induces the activation of microglial cells and overproduction of inflammatory factors such as oxidative stress products,[IL]-6, [IL]-1β, and [TNF]-ɑ, ultimately leading to neuronal injury even ensuing cognitive dysfunction. We speculated that MKP-1-induced activation of hippocampal microglia and release of inflammatory factors may contribute to the development of depression under chronic stress.

The purpose of the current study was to examine if aberrant MKP-1 activity contributes to depression-like behavior. To this end, we compared depression-like behaviors and expression levels of microglial activation and neuroinflammatory markers among three animal models: (1) a rat model of depression induced by chronic unpredictable mild stress (CUMS), (2) rats overexpressing MKP-1 by adeno-associated virus (AAV) infection, and (3) CUMS-exposed rats underexpressing MKP-1 due to AAV-induced knockdown. Animals were examined for depression-like behaviors using the sucrose preference test, open field test, and forced swimming test. In addition, the hippocampal expression levels of MKP-1, phosphorylated (activated) MAPKs (p-ERK, p-p38, p-JNK), and inflammation-related indicators were measured by Western blotting, real-time PCR, and immunofluorescence assays. We demonstrate that MKP-1 overactivation in hippocampus is associated with microglial activation, neuroinflammation, and depression-like behaviors following chronic stress, while MKP-1 knockdown can protect rats from CUMS-induced neuroinflammation and depression-like behavioral changes.

## Material and methods

### Animals

All experimental procedures were approved by the Henan Key Laboratory of Biopsychiatry Committee (approval number HMH. No. 20190806AEC018) and conducted according to the guidelines of the Chinese National Science Academy for the use and care of experimental animals. The researchers conducting experimental interventions and sample processing were unaware of the grouping situation. Procedures were designed to minimize the number of animals used and their suffering. Adult male Sprague-Dawley rats (Charles River, Beijing, China) weighing 180–220 g were housed under controlled temperature (24 ± 1 °C), controlled humidity (50% ± 10%), and a regular light–dark cycle (lights on at 8:00 a.m., lights off at 8:00 p.m.) and with free access to water and food except during some experimental sessions as indicated. Rats were allowed to acclimate for 1 week before surgery or experiments.

### Experiment design

The study consisted of three experimental phases using different animal models.

**Fig. 1:**
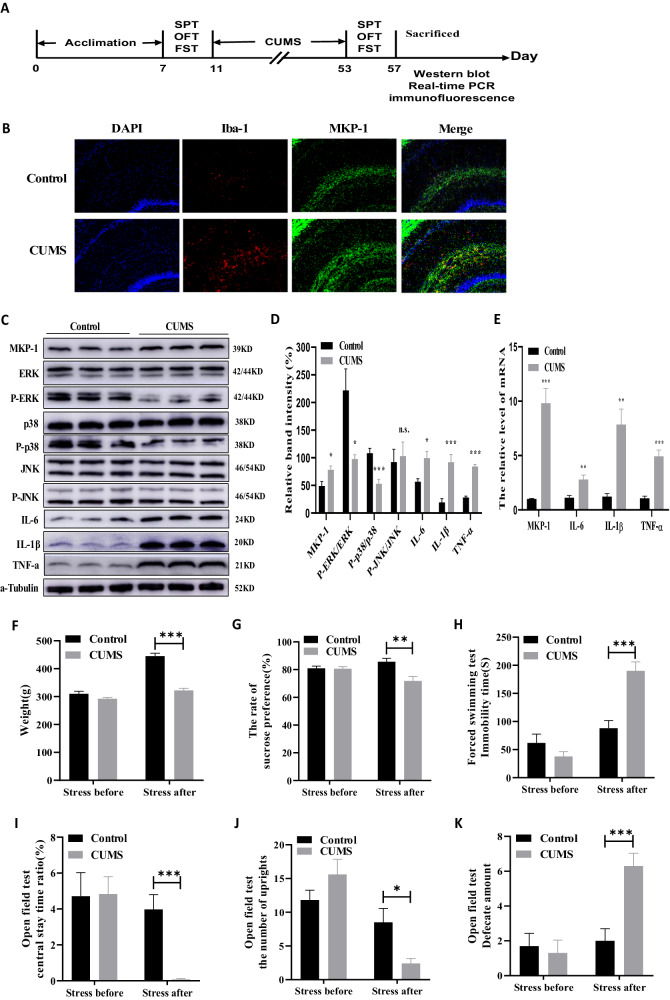
Chronic unpredictable mild stress (CUMS) upregulates MKP-1 and pro-inflammatory cytokines in the hippocampus, increases hippocampal microglial number, and promotes depression-like behaviors in rats. **A** Timeline of the experimental procedure. **B** Representative fluorescence image of MKP-1 and microglial marker Iba-1 immunoexpression in the hippocampus of sham control (CON) and CUMS-exposed rats (*N* = 3 rats per treatment group). **C** Representative immunoblots of MKP-1, ERK, p-ERK, p-38, p-p38, JNK, p-JNK, [IL]-6, [IL]-1β, and [TNF]-ɑ protein expression in the hippocampus after CUMS. **D** CUMS increased the expression levels of MKP-1, [IL]-6, [IL]-1β, and [TNF]-ɑ but decreased p-ERK/ERK and p-p38/p38 in the hippocampus (**C** and **D**: Data presented as mean ± SEM of *N* = 6 rats per group. **P < 0.01 by independent samples *t*-test, n.s., not significant). **E** CUMS increased the mRNA expression levels of MKP-1, [IL]-6, [IL]-1β, and [TNF]-ɑ in the hippocampus (Data presented as mean ± SEM of *N* = 6 rats per group. **P < 0.01 by independent samples *t*-test). **F** CUMS exposure reduced mean body weight and sucrose preference compared to control rats. **H**–**K** CUMS prolonged immobility time in the force swimming test (**H**), decreased central time ratio in the open field test (**I**), and reduced rearing frequency (**J**). **K** CUMS also increased defecation frequency in the open field test (**F**–**K**: Data presented as mean ± SEM of *N* = 10 rats per group. *P < 0.05, **P < 0.01, ***P < 0.001 by repeated measures analysis of variance).

In Experiment 1, naïve SD rats were randomly assigned to a control group (*N* = 10) and a chronic unpredictable stress group (*N* = 10). Rats in the chronic unpredictable stress (CUMS) group were subjected to various daily stressors for 42 days while control rats received daily handling (Fig. 1A). The CUMS protocol is described in detail in the section “Chronic unpredictable mild stress (CUMS) procedure”. Depressive-like behaviors were measured using the open field test (OFT), forced swimming test (FST), and sucrose preference test (SPT) before and after CUMS or control handling. Rats were sacrificed 24 h after the last behavioral test. The hippocampal expression levels of MKP-1, MAPK isoforms extracellular regulated kinase (ERK), phosphorylated (p)-ERK, c-Jun N-terminal kinase (JNK), p-JNK, p38 MAPK, p-p38 MAPK, [IL]-1β,[IL]-6, and [TNF]-α were estimated by western blotting (described in the section “Western blot analysis”) and real-time PCR (see the section “Real-time PCR”), while co-expression of MKP-1 with the microglial marker IBA-1 was examined in brain slices by immunofluorescence staining (see the section “Immunofluorescence”).

**Fig. 2:**
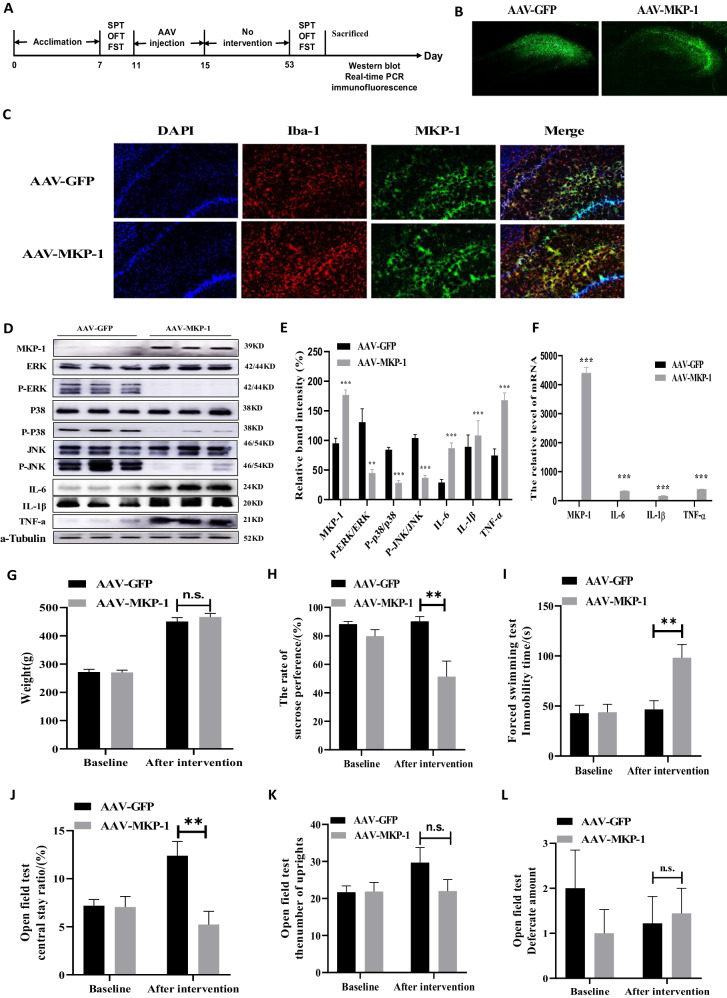
Overexpression of MKP-1 in the hippocampus induces microglial proliferation, pro-inflammatory cytokine production, and depression-like behaviors in unstressed rats. **A** Timeline of the experimental procedure. **B** Representative fluorescence image of adeno-associated virus (AAV) injection sites in the hippocampus. **C** Representative immunofluorescence images of MKP-1 and Iba-1 expression in hippocampal sections from the AAV-GFP (control) and AAV-GFP-MKP-1 (MKP-1 overexpression) groups (*N* = 3 rats per group). **D** Representative immunoblots of MKP-1, ERK, p-ERK, p-38, p-p38, JNK, p-JNK, [IL]-6, [IL]-1β, and [TNF]-ɑ protein expression in the hippocampus of AAV-GFP and the AAV-GFP-MKP-1 group rats. **E** The expression levels of MKP-1, [IL]-6, [IL]-1β, and [TNF]-ɑ were significantly increased, while p-ERK/ERK, p-p38/p38, and p-JNK/JNK expression were significantly decreased in the AAV-GFP-MKP-1 group (**D** and **E**: Data presented as mean ± SEM of *N* = 6 rats per group. **P < 0.01 by independent samples *t*-test, n.s., not significant). **F** Injection of MKP-1 overexpression vector increased the expression of MKP-1, [IL]-6, [IL]-1β, and [TNF]-ɑ mRNA in the hippocampus (Data presented as mean ± SEM of *N* = 3 rats per group. **P < 0.01 by independent samples *t*-test, n.s., not significant). **G** MKP-1 overexpression did not affect body weight. **H**–**J** MKP-1 overexpression reduced sucrose preference (**H**), enhanced immobility time in the forced swimming test (**I**), and reduced the central time ratio in the open field test (**J**). **K**, **L** MKP-1 overexpression did not affect rearing frequency (**K**) or defecation frequency (**L**) in the open field test (**G**–**L**: Data presented as mean ± SEM of *N* = 9 rats per group. ***P < 0.001 by repeated measures analysis of variance).

Experiment 2: A second group of naïve rats was randomly divided into an AAV-control microinjection group and an AAV-MKP-1 microinjection group (*N* = 9). Rats received bilateral injections of the AAV virus in the hippocampus (Fig. 2A). Six weeks later, depressive-like behaviors and gene or protein expression changes were assessed using the same methods as in Experiment 1.

**Fig. 3:**
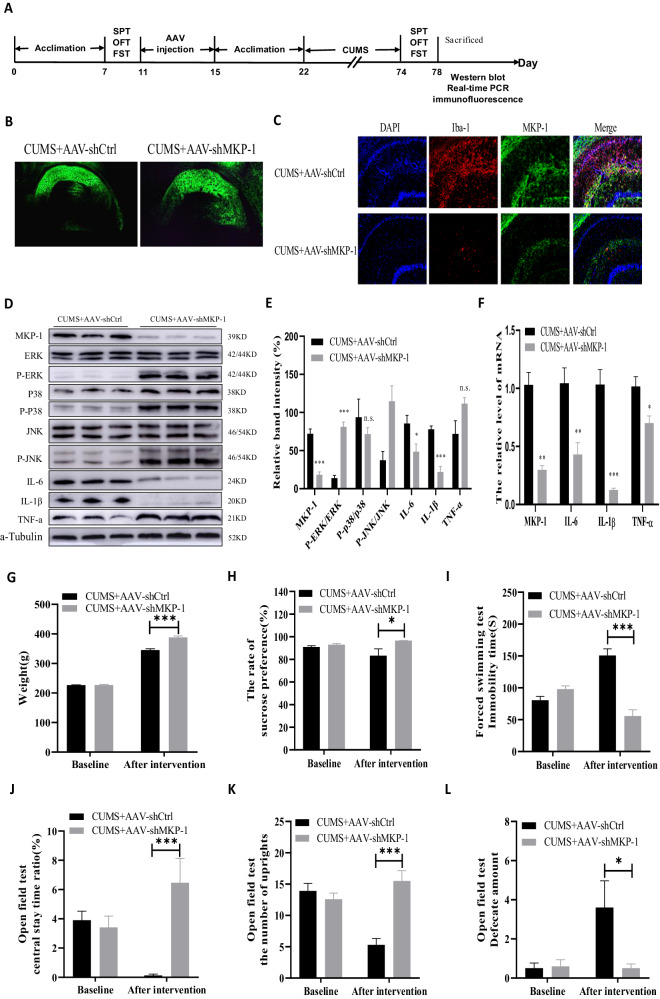
Knockdown of MKP-1 in the hippocampus protects against CUMS-induced neuroinflammation and depression-like behaviors. **A** Timeline of the experimental procedure. **B** Representative fluorescence image of injection sites in the hippocampus. **C** Representative fluorescence images of MKP-1 and Iba-1 expression in the hippocampus of CUMS + AAV-Ctrl (control) and CUMS + AAV-shMKP-1 (knockdown) groups (*N* = 3 rats per group). **D** Representative immunoblots of MKP-1, ERK, p-ERK, p-38, p-p38, JNK, p-JNK, [IL]-6, [IL]-1β, and [TNF]-ɑ protein expression in the hippocampus. **E** p-ERK/ERK, p-JNK/JNK, and [TNF]-ɑ expression levels were significantly increased, while MKP-1, [IL]-6, and [IL]-1β expression levels were significantly decreased by MKP-1 knockdown (Data presented as mean ± SEM of *N* = 7 rats per group. **P < 0.0 by independent samples *t*-test, n.s., not significant). **F** Hippocampal MKP-1 knockdown effectively reversed the CUMS-induced increases in [IL]-6 and [IL]-1β mRNA expression (Data presented as mean ± SEM of *N* = 6 rats per group. **P < 0.01 by *t-*test). **G** Hippocampal MKP-1 knockdown did not affect body weight. **H** and **I** Hippocampal MKP-1 knockdown effectively reversed the CUMS-induced reduction in sucrose preference (**H**) as well as the CUMS-induced increase in immobility time during the forced swimming test (**I**). MKP-1 overexpression also reversed the CUM-induced reduction in central time ratio (**J**), the increase in rearing frequency (**K**), and (**L**) the increase in defecation frequency during the open field test (**G**–**L**: Data presented as mean ± SEM of *N* = 10 rats per group. ***P < 0.001 by repeated measures analysis of variance).

Experiment 3: A third rat group was randomly into CUMS + AAV-control and CUMS + Down-MKP-1 groups (*N* = 10). Rats were subjected to CUMS for 42 days as detailed in the section “Chronic unpredictable mild stress (CUMS) procedure” following injection of the AAV virus (Fig. 3A). Six weeks after the final CUMS exposure, depressive-like behaviors, and gene or protein expression changes were assessed using the same methods as in Experiment 1.

### AAV vectors injection

Adeno-associated virus-9 (AAV-9) vectors for MKP-1 overexpression (AAV-GFP-MKP-1), MKP-1 knockdown (AAV-shMKP-1), and corresponding controls (AAV-GFP and scrambled AAV-shCtrl, respectively) were obtained from Shanghai Genechem Co., Ltd. (Shanghai, China). The virus titer was 1E + 13 v.g./mL. After adaptation to the laboratory environment, animals were anaesthetized with 3% pentobarbital sodium (3 mL/kg body weight, i.p.) and infused with AAVs into the bilateral hippocampus CA1 under stereotaxic guidance (anterior–posterior position −3.0 mm, medial–lateral position ±3 mm, −3.6 mm dorsoventral from the bregma) and CA3 (anterior–posterior position −4.5 mm, medial–lateral position ±4.8 mm, −3.6 mm dorsoventral from the bregma) for 10 min at 0.1 μL/min (1 μL injection per site) using a micro-syringe. Needles remained in place for an additional 10 min to allow diffusion of the vector. The shMKP-1 targeting sequence used was CCATGATTCCTTCATATTTGC.

### Chronic unpredictable mild stress (CUMS) procedure

The CUMS model was established as described previously [21, 22]. Rats were housed individually in a separate room and randomly subjected to 2 or 3 of the following stressful stimuli daily for 6 weeks: electric foot shocks (0.65 mA, 20 times, 5 min), tail pinch (5 min), ice water swimming (4 °C, 5 min), reversed light/dark cycle (24 h), food deprivation (24 h), water deprivation (24 h), restraint (6 h), cage tilting (45°, 12 h), damp bedding (200 mL water, 200 g bedding, 24 h), crowded housing (6 or 7 animals per cage, 24 h), empty drinking water bottle (2 h), and white noise (85 dB, 6 h). The same stressor was not applied on 2 consecutive days to avoid habituation. After 6 weeks of CUMS, behavioral tests were performed in the order SPT, OFT, and FST.

### Behavioral tests

All behavioral tests were conducted during the light phase. Rats were transferred to the experimental room 2 h prior to testing for acclimation. Behavioral tests were performed according to previous reports with some modifications [10].

#### Sucrose preference test (SPT)

Two sucrose preference tests were conducted, a test of baseline sucrose preference and a second test for changes in sucrose preference due to CUMS and/or manipulation of hippocampal MKP-1 expression. Each test was preceded by an adaptation phase. On the first day of adaptation, each rat was individually placed in a cage with two similar bottles filled with 1% sucrose solution, of which one was exchanged with another containing water on the second day. The position of the water and sucrose bottles were switched after 12 h to avoid any position preference. On the third day of the adaptation phase, rats had no access to food or water. Rats were then transferred individually to the experimental room for a 12-h test of sucrose preference in which two identical bottles, one containing 1% sucrose solution (w/v) and the other containing tap water, were presented simultaneously. The positions of the bottles were switched after 6 h. Sucrose preference (%) was calculated as

sucrose intake (mL)/[sucrose intake (mL) + water intake (mL)] × 100%.

#### Open field test (OFT)

The open field test was performed in a chamber (length 100 cm × width 100 cm × height 40 cm) under dim room lighting between 08:00 and 12:00. Rats were placed individually in the central area and allowed to explore freely for 5 min. The behavior of each rat was recorded with a video camera and movement was traced using Smart Version 3.0. Rearing and defecation frequencies were also calculated manually during each test. Between sessions, the apparatus was wiped with 70% ethanol.

#### Forced swimming test (FST)

In brief, an individual rat was placed gently in a glass cylindrical container (30 cm in diameter, 80 cm deep) filled to a depth of 40 cm with water (24 ± 1 °C) and observed for 6 min. Immobile time was defined as the time spent free-floating without struggling. The duration of immobility was recorded by 2 observers blind to the group identity over the final 5 min. The water was replaced for each testing session.

### Western blot analysis

Each microgram of hippocampal protein was added to 10 μL of a mixture with magnetic beads, which was a mixture of radioimmunoprecipitation assay (RIPA) lysis buffer (Beyotime Biotechnology) and phenylmethanesulfonyl fluoride (PMSF) (100:1), incubation for 30 min on ice, and centrifugation at 1200×*g* for 15 min at 4 °C. Total supernatant protein concentration was assessed using a bicinchoninic acid (BCA) kit (Beyotime Biotechnology). Equal amounts of protein from each sample (20 g) were separated by sodium dodecyl sulfate-polyacrylamide gel electrophoresis (SDS–PAGE) and transferred onto polyvinylidine difluoride (PVDF) membranes (Millipore, USA). The membranes were blocked in 10% fat-free milk in the tris-buffered saline containing 0.05% Tween 20 (TBST) for 2 h at room temperature and incubated overnight at 4 °C with anti-MKP-1 (1:2000, Abcam, ab195261), p-ERK 1/2 (1:2000, Cell Signaling Technology, 4370s), ERK (1:2000, Cell Signaling Technology, 4695s), p-SAPK/JNK (1:2000, Cell Signaling Technology, 4668s), SAPK/JNK (1:2000, Cell Signaling Technology, 9252s), p38 MAPK (1:2000, Cell Signaling Technology, 8690s), p-p38 MAPK (1:2000, Cell Signaling Technology, 4511s),[IL]-6 (1:1000, Proteintech, 21865-1-AP), [IL]-1β (1:1000, Cloud-Clone, MAA 563Ra 21), [TNF]-ɑ (1:500, Affinity Biosciences, Ab-AF7014), and/or α-tubulin (1:2000, Proteintech, 66031-1-Ig) as the gel loading control. The next day, membranes were washed three times with TBST, incubated with horseradish peroxidase (HRP)-conjugated secondary antibody for 2 h at room temperature, and rewashed 3 times with TBST. Target protein bands were visualized using an ECL reagent (Millipore) and the ECL signal was captured using an ImageQuant LAS4000 mini image analyzer (GE Healthcare). Band density was quantified using ImageJ software (NIH, Bethesda) as an estimate of protein expression level.

### Real-time PCR

Total RNA was extracted from dissected hippocampal tissues using TRIzol reagent (Invitrogen, Thermo Fisher Scientific Inc.) and quantified by absorbance at 260 nm. Quantitative real-time PCR (RT-PCR) was conducted using the RNAsimple Total RNA kit (Tiangen Biotech Co., Ltd.) on a QuantStudio™ 6 Flex Real-Time PCR System. Expression of GAPDH mRNA was measured as the internal standard. The primer sequences are as follows: MKP-1 Forward GGGCCCAGTGGAGATCCTGTCC, MKP-1 Reverse AGCAGTGATACCCAAGGCGTCG,[IL]-6 Forward AAGGACCAAGACCATCCAAC,[IL]-6 Reverse ACCACAGTGAGGAATGTCCA, [IL]-1β Forward GCATCCAGCTTCAAATCTCA, [IL]-1β Reverse ACGGGCAAGACATAGGTAGC, [TNF]-α Forward GAGATGTGGAACTGGCAGAG, [TNF]-α Reverse AGCAGGAATGAGAAGAGGCT, GAPDH Forward GAAGGTCGGTGTGAACGGAT, GAPDH Reverse ACCAGCTTCCCATTCTCAGC.

### Immunofluorescence

Immunofluorescence staining of the hippocampus was conducted on 3 rats per treatment group. Rats were anesthetized by intraperitoneal injection of 10% chloral hydrate (4 mg/kg) and perfused through the heart with 4% paraformaldehyde (PFA) in 0.1 M sodium phosphate buffer. Brains were post-fixed for 48 h in the same paraformaldehyde solution and then reserved in 10%、15%、30% sucrose until the brain tissue settled to the bottom of the bottle. The brain was then sectioned at 8 μm in the coronal plane using a freezing microtome. Sections were heated three times at 5-min intervals in boiling 1% Citrate-EDTA Antigen Retrieval Solution (Beyotime Biotechnology), washed 3 times in phosphate-buffered saline (PBS), permeabilized with 1% Triton X-100, and blocked with 10% goat serum for 30 min at room temperature. Sections were then incubated in primary antibodies targeting the microglial marker binding adaptor molecule-1 (1ba-1, 1:250, Abcam, ab178846) and mitogen-activated protein kinase phosphatase-1 (MKP-1,1:100, Santa Cruz, sc373841) overnight at 4 °C, washed three times in PBS, incubated with Alexa Fluor 647/488-AffiniPure secondary antibodies (1:1000, Jackson ImmunoResearch 111-605-003;115-545-003) for 1 h at 37 °C, and counterstained with 4′, 6-diamidino-2-phenylindole (DAPI) for 5 min. Stained sections were viewed under a fluorescence microscope (Nikon, Eclipse Ci-L, Japan). Then, we visualized as many immunostained cells as possible, and then conducted background subtraction (Image-Pro Plus).

### Statistical analysis

Statistical analyses were performed using GraphPad Prism 8 and SPSS 22.0 statistical packages. Western blot, RT-PCR, and immunostaining results were compared by independent samples Student’s *t*-test, while group body weight and behavioral results were compared by repeated measures ANOVA. The data are presented as mean ± standard error of the mean (SEM). Values of *P* < 0.05 were considered statistically significant for all tests.

## Results

### Chronic stress-induced depressive-like behaviors, reduced hippocampal MPK-1 expression, and upregulated inflammatory marker expression in rats

**Fig. 4:**
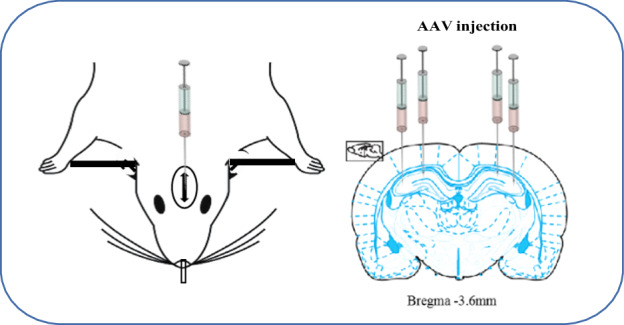
Schematic diagram of the virus injection protocol. Adeno-associated virus, serotype 9 (AAV-9) expressing green fluorescent protein (GFP) or GFP plus pGCSIL-F (4185-4165: CCATGATTCCTTCATATTTGC) was injected into the bilateral hippocampus under stereotaxic guidance. The virus titer was 1E + 13 v.g./mL.

Chronic stress plays an important role in the etiology of depression [23] and CUMS is a well-validated animal model of stress-induced depression [24]. Behavioral tests were conducted before and after CUMS, subsequent western blotting, RT-PCR, and immunofluorescence analyses of microglial activation and neuroinflammation in the hippocampus (Fig. 4).

Rats allocated to receive CUMS or control treatment did not differ significantly in baseline body weight and demonstrated no baseline differences in SPT, OFT, and FST behavioral metrics. Compared to the control group, however, rats exposed to CUMS exhibited significantly reduced body weight [*F*(1,18) = 84.082, *P* = 0.000, Fig. 1F], reduced sucrose preference [*F*(1,18) = 11.402, *P* = 0.003, Fig. 1G], and greater immobility time in the FST [*F*(1,18) = 23.681, *P* = 0.000, Fig. 1H] as well as more frequent defecation [*F*(1,18) = 18.068, *P* = 0.000, Fig. 1K], reduced central stay time ratio [*F*(1,18) = 22.486, *P* = 0.000, Fig. 1I], and rearing frequency [*F*(1,18) = 7.700, *P* = 0.012, Fig. 1J] in the OFT. Thus, CUMS-exposed rats exhibited anhedonia, behavioral despair, and anxiety, the core symptoms of the depressive rat phenotype.

In addition to these behavioral signs of depression, western blotting (Fig. 1C, D) revealed significant upregulation of MKP-1 (*t*_(10)_ = 2.698, *P* = 0.022),[IL]-6 (*t*_(10)_ = 3.156, *P* = 0.010), [IL]-1β (*t*_(10)_ = 4.582, *P* = 0.001), and [TNF]-ɑ (*t*_(10)_ = 11.900, *P* = 0.000), but significant downregulation of p-ERK/ERK (*t*_(10)_ = 3.119, *P* = 0.011) and p-p38/p38 (*t*_(10)_ = 4.632, *P* = 0.001) in the hippocampus after CUMS. In contrast, p-JNK/JNK expression was not changed in the hippocampus after CUMS compared to baseline (*t*_(10)_ = 0.312, *P* = 0.761). Similarly, real-time PCR revealed (Fig. 1E) significant upregulation of MKP-1 mRNA (*t*_(10)_ = 6.458, *P* = 0.000),[IL]-6 mRNA (*t*_(10)_ = 3.633, *P* = 0.004), [IL]-1β mRNA (*t*_(10)_ = 4.586, *P* = 0.001), and [TNF]-ɑ mRNA (*t*_(10)_ = 6.318, *P* = 0.000) following CUMS, indicating that the observed behavioral signs of depression were accompanied by suppressed ERK and p38 MAPK signaling and elevated inflammatory signaling in the hippocampus.

Furthermore, immunofluorescence showed that MKP-1was co-localized with the microglia-specific marker Iba-1 after CUMS (Fig. 1B). These results suggest that chronic stress may activate the inflammatory response in hippocampal microglia by activating MKP-1 and MAPKs signaling pathways.

### MKP-1 overexpression in the hippocampus induced depressive-like behaviors in unstressed rats

As CUMS increased MKP-1 expression in the hippocampus, we tested if experimental overexpression of MKP-1 in the rat hippocampus can also induce neuroinflammation and behavioral signs of depression (see Fig. 2A for the experimental timeline). Adeno-associated virus vectors selectively expressing MKP-1 plus GFP as an expression marker (AAV9-GFP-MKP-1) or GFP alone as a control (AAV9-GFP) were infused into the bilateral hippocampi of non-stressed rats using stereotaxic guidance. After 35 days, GFP expression was observed in brain slices to confirm successful expression (Fig. 2B). Expression of MKP-1 was significantly higher in the AAV9-GFP-MKP-1 infusion group at both the protein level as indicated by western blotting (*t*_(10)_ = −6.705, *P* = 0.000, Fig. 2D, E) and the mRNA level as indicated by real-time PCR (*t*_(10)_ = 23.730, *P* = 0.000, Fig. 2F).

Compared to the AAV9-GFP (control) group, rats infused with AAV9-GFP-MKP-1 and overexpressing MKP-1 in the hippocampus demonstrated significantly reduced sucrose preference [*F*(1,16) = 11.458, *P* = 0.004, Fig. 2H] and longer immobility time in the FST [*F*(1,16) = 10.583, *P* = 0.005, Fig. 2I] as well as reduced central area time [*F*(1,16) = 12.657, *P* = 0.003, Fig. 2J], greater rearing frequency [*F*(1,16) = 2.216, *P* = 0.156, Fig. 2K], and higher defecation frequency [*F*(1,16) = 0.074, *P* = 0.788, Fig. 2L] in the OFT. These results suggest that overexpression of MKP-1 in the hippocampus can induce depressive-like behaviors even in the absence of stress.

### MKP-1 overexpression in the hippocampus increased microglial number and expression of pro-inflammatory cytokines by suppressing MAPK pathways

In addition to exhibiting the core behavioral phenotypes of rat depression models, rats overexpressing MKP-1 in the hippocampus showed elevated expression levels of the inflammatory mediators [IL]-6 (*t*_(10)_ = -5.490, *P* = 0.000) and [TNF]-ɑ (*t*_(10)_ = -5.630, *P* = 0.000) but reduced p-ERK/ERK (*t*_(10)_ = 3.648, *P* = 0.012), p-p38/p38 (*t* = 10.016, *P* = 0.000), and p-JNK/JNK (*t*_(10)_ = 9.634, *P* = 0.000) as measured by Western blotting (Fig. 2D, E). Similarly, real-time PCR (Fig. 2F) showed that MKP-1 overexpression was associated with upregulation of[IL]-6 mRNA (*t*_(10)_ = 48.160, *P* = 0.000), [IL]-1β mRNA (*t*_(10)_ = 27.270, *P* = 0.000), and [TNF]-ɑ mRNA (*t*_(10)_ = 176.900, *P* = 0.000). In contrast, expression of [IL]-1β was not altered by MKP-1 overexpression (*t*_(10)_ = −0.590, *P* = 0.568), in accordance with measurements following after CUMS (*t*_(10)_ = 0.463, *P* = 0.652).

Furthermore, expression of the microglial cell marker Iba-1 was enhanced in hippocampal sections from the AAVp-GFP-MKP1 group compared to the AAV-GFP group (Fig. 2C). These results suggest that overexpression of MKP-1 and concomitant negative regulation of MAPK signaling in the hippocampus may induce the proliferation and activation of microglia, resulting in enhanced release of inflammatory cytokines, neuroinflammation, and depression-like behaviors.

### Hippocampal MKP-1 knockdown protects against CUMS-induced depressive-like behaviors

We then examined the effect hippocampal MKP-1 knockdown on CUMS-induced depression-like behaviors (Fig. 3A shows the experimental timeline). Rats were injected with either AAV9-GFP-shMKP-1 or empty AAV9-GFP-shCtrl and the efficacy of knockdown was first confirmed by both western blotting (*t*_(10)_ = 7.294, *P* = 0.000) and real-time PCR (*t*_(10)_ = 23.730, *P* = 0.000) (Fig. 3B and D, F). One week after the AAV-9 infusion, rats were subjected to the CUMS procedure. Repeated measures ANOVA showed that MKP-1 knockdown significantly reversed the CUMS-induced reduction in sucrose consumption [*F*(1,18) = 4.762, *P* = 0.043, Fig. 3H] and increase in FWT immobility time [*F*(1,18) = 43.457, *P* = 0.000, Fig. 3I] as well as the CUMS-induced decrease in central stay time ratio [*F*(1,18) = 14.205, *P* = 0.001, Fig. 3J], increased rearing frequency [*F*(1,18) = 27.984, *P* = 0.000, Fig. 3K], and increased defecation frequency [*F*(1,18) = 5.002, *P* = 0.038, Fig. 3L] in the OFT.

### Hippocampal MKP-1 knockdown suppresses CUMS-induced neuroinflammation by disinhibition of MAPK signaling

Our findings that hippocampal MKP-1 overexpression induced both depression-like behaviors and local neuroinflammation suggested that MKP-1 knockdown reversed CUMS-induced depression-like behaviors by quelling the associated neuroinflammatory response. Consistent with this speculation, MKP-1 knockdown reversed the CUMS-induced increases in hippocampal [IL]-6 (*t*_(10)_ = 2.506, *P* = 0.031) and [IL]-1β (*t*_(10)_ = 6.651, *P* = 0.000) as well as [IL]-6 mRNA (*t*_(10)_ = 3.674, *P* = 0.004) and [IL]-1β mRNA (*t*_(10)_ = 7.079, *P* = 0.000). In contrast, there was no significant effect on CUMS-induced [TNF]-ɑ elevation at either the protein level (*t*_(10)_ = 2.106, *P* = 0.061) or mRNA level (*t*_(10)_ = 1.552, *P* = 0.152).

As MAPK pathways regulate the inflammatory response [16], we also examined if MKP-1 knockdown reversed the effects of CUMS on MAPK activation (Fig. 3D, E). Indeed, hippocampal MKP-1 knockdown prevented both the CUMS-induced reduction in p-ERK/ERK (*t*_(10)_ = 9.515, *P* = 0.000) and p-JNK/JNK (*t*_(10)_ = 3.337, *P* = 0.008), but not the CUMS-induced decrease in p-p38/p-38 (*t*_(10)_ = 0.881, *P* = 0.399). Finally, immunofluorescence staining of hippocampal sections also revealed that MKP-1 knockdown reversed the CUMS-induced increase in microglial cell number (Fig. 3B). Collectively, these data suggest that MKP-1 knockdown and concomitant disinhibition of MAPK signaling prevents stress-induced activation of rat hippocampal microglia, ensuing neuroinflammation, and consequent depression-like behaviors.

## Discussion

Chronic stress-induced mental disorders are strongly associated with neuroinflammation in the hippocampus [25], and MKP-1 is a crucial regulator of microglial activation and inflammation in the brain [26]. In the current study, we show that chronic stress significantly increases MKP-1 expression in the rat hippocampus and that hippocampal overexpression alone is sufficient to elicit local neuroinflammation and multiple depression-like behaviors. Most importantly, we found that hippocampal MKP-1 knockdown using a targeted shRNA and consequent disinhibition of MAPK signaling effectively protected against CUMS-induced neuroinflammation and suppressed depressive-like behaviors.

MKP-1 is a highly conserved protein strongly implicated in the development of several organic diseases [27], including cancer, rheumatoid arthritis (RA), and asthma, and more recent studies have suggested that MKP-1 dysregulation also contributes to neuroinflammation and neuropsychiatric diseases [28–33]. Thus, modulating brain MKP-1 activity may be a potential treatment for psychiatric disorders. MKP-1 is an important feedback control mechanism that limits MAPK signaling and subsequent target gene expression. To the best of our knowledge, this study shows for the first time that MKP-1 expression and MAPK pathway activity are altered in the CUMS model of depression. Briefly, we found that hippocampal MKP-1 expression was significantly increased by CUMS and that this increase was negatively associated with ERK and p38 MAPK phosphorylation/activation and positively associated with reduced sucrose preference, a behavioral endophenotype of depression-related anhedonia. Alternatively, there was no change in hippocampal p-JNK/JNK, suggesting that ERK and p38 MAPK pathways act to preserve normal neuroinflammatory levels, neural processing, and behavioral function under stress. However, further study is needed to determine the specific functions of ERK, p38 MAPK, and JNK in the etiology of depression. Given this uncertainty, the application of MKP-1 inhibitors may be a better approach to improve the neurological dysfunction and damage induced by stress. Accordingly, in future studies, we will examine if pharmacological MKP-1 inhibitors can also mitigate stress-induced neuroinflammation and depressive-like behavior.

Both CUMS and MKP-1 overexpression in the hippocampus induced multiple depressive-like behaviors as revealed by the sucrose preference, forced swimming, and open field tests, consistent with previous findings [10, 34]. The open field test is generally used to assess state and trait depressive-like behaviors [35] and both CUMS-exposed rats and MKP-1-overexpressing rats demonstrated relatively reduced activity in the center of the field, a hallmark of decreased interest and adaptability of rodents to new environments in this test, while MKP-1 knockdown in the hippocampus mitigated this and other behavioral signs of elevated depression. These results provide compelling evidence that MKP-1 hyperactivity and ensuing MAPK signaling insufficiency contribute to the pathogenesis of chronic stress-induced depression.

Emerging evidence suggests that neuroinflammation is a major pathomechanism underlying MDD onset and progression [36, 37]. For instance, elevated proinflammatory cytokine levels have been reported in the peripheral blood and cerebrospinal fluid of patients with depression [13], and anti-inflammatory drugs have been found to both relieve depression to a certain extent and reduce the incidence of depression [38]. Nonetheless, the precise mechanisms linking environmental stress and neuroinflammatory to depression remain unclear Excessive activation of microglia can effectively facilitate the neuroinflammatory response [3], by releasing pro-inflammatory factors [39]. A growing number of studies have also reported that nascent neuronal damage can lead to an increase in inflammatory factors [34] and that early-life inflammation causes dysregulation of microglial engulfment capacity, ultimately leading to depression-like symptoms [37]. We found that stress-induced abnormal MKP-1 elevation in hippocampal neurons while MKP-1 knockdown reduced stressed-associated neuroinflammation and reversed stress-induced downregulation of ERK and p38 MAPK signaling pathways, suggesting that neuroinflammation increases depression risk by upregulating MKP-1 and leading to insufficient MAPK signaling, which is necessary for neuronal survival and adaptive plasticity under stress. To directly demonstrate the contribution of MAPK signaling insufficiency to depression pathogenesis, in future studies we will examine the effects of hippocampal ERK and p38 knockdown on neuroinflammation and depressive behaviors following CUMS.

While there is accumulating evidence that MAPK signaling pathways are involved in depression, the specific contributions of ERK, p38 MAPK, and JNK [40] remain unclear. In contrast to the current findings, a non-selective MAPK inhibitor was reported to alleviate depression in rodents [41] while sustained activation was found to promote the inflammatory responses of microglia activated under various stressors, including chronic mild stress [42]. ERK can activate substrates involved in cell growth, apoptosis, differentiation, and proliferation [43, 44], and Wang and colleagues [45] reported marked downregulation of ERK signaling in the prefrontal cortex and hippocampus of depressed humans and various animal models of chronic depression. Further, these effects were reversed by the antidepressants amitriptyline and fluoxetine. Thus, ERK signaling activity may be a major driver of neurological processes that protect against stress-induced dysfunction and behavioral depression. Consistent with this notion, we found that CUMS-induce depression-like behaviors were associated with lower ERK phosphorylation, while MKP-1 knockdown both reversed this p-ERK downregulation and depression-like behaviors.

The p38 MAPK isoform is highly expressed in brain regions critical for learning and memory where it is known to coordinate molecular responses to cellular stress [46]. In previous studies of anxiety and depression models, however, the changes in p38 were inconsistent. Fan and colleagues [47] found that environmental stress activated the p38 signaling pathway, which in turn induced microglial activation and the release of pro-inflammatory factors. In contrast, Asih and colleagues [48] found that p38α deletion in neurons increased anxiety in the open field and elevated plus maze. Our findings are more consistent with the latter study, as p-p38 was significantly reduced in rats exposed to CUMS and overexpressing MKP-1, suggesting that deficient p38 signaling is involved in the development of depression. Indeed, the knockdown of MKP-1 not only suppressed the depression-like behaviors of CUMS-exposed rats, but it also reversed the associated decrease in p38 and increased total p38 expression.

Like p38 MAPK, c-Jun amino-terminal kinase (JNK) is an important regulator of inflammation and stress responses implicated in central nervous system diseases [49]. Preventing JNK activation was reported to alleviate anxiety and depressive behavior [50]. Also, the JNK inhibitor SP600125 ameliorated lipopolysaccharide (LPS)-induced depressive-like behaviors and reduced the levels of pro-inflammatory cytokines in brain regions relevant to MDD [49]. However, we found no change in hippocampal JNK expression following CUMS, but significantly reduced p-JNK concomitant with MKP-1 overexpression, a change reversed by MKP-1 knockdown. The changes in JNK activity and contributions to disease progression may differ among depression models or brain regions. Nonetheless, the effects of MKP-1 on ERK expression and activity have been greater and more consistent across studies, so ERK stabilization may be a more promising treatment strategy for MDD.

## Conclusion

This study demonstrates that overexpression of MKP-1 and ensuing ERK signaling insufficiency are seminal events linking environmental stress to depression-like disorders in rats. Blockade of MKP-1 may thus be an effective treatment strategy for clinical depression.

## Data Availability

All data needed to evaluate the conclusions in the paper are presented in the paper.
